# Interferon-induced transmembrane protein 1 (IFITM1) is required for the progression of colorectal cancer

**DOI:** 10.18632/oncotarget.13325

**Published:** 2016-12-11

**Authors:** Novita Ita Sari, Ying-Gui Yang, Lan Thi Hanh Phi, Hyungjoo Kim, Moo Jun Baek, Dongjun Jeong, Hyog Young Kwon

**Affiliations:** ^1^ Soonchunhyang Institute of Medi-bio Science (SIMS), Soonchunhyang University, Republic of Korea; ^2^ Soonchunhyang Medical Science Research Institute, College of Medicine, Soonchunhyang University, Republic of Korea; ^3^ Department of Surgery, Department of Pathology, College of Medicine, Soonchunhyang University, Republic of Korea

**Keywords:** IFITM1, colorectal cancer, prognosis, metastasis

## Abstract

Interferon-induced transmembrane protein 1 (IFITM1) has been shown to be implicated in multiple cancers, yet little is known about biological significance of IFITM1 in colorectal cancer. Here, we show that IFITM1 is highly expressed in metastatic colorectal cancer cell lines as well as colorectal patient-derived tumor samples, and its expression is associated with a poor prognosis of the disease. Also, IFITM1 depletion resulted in a significant reduction in the mobility of cancer cell lines, whereas ectopic expression of IFITM1 promoted the migration of cancer cells. Epithelial-mesenchymal transition (EMT) signature was dysregulated by both loss and gain of function of IFITM1, which was partially reverted by Caveolin-1 (CAV1). Therefore, these results suggest that IFITM1 may be a prognostic marker and an attractive target to achieve better therapeutic outcomes in colorectal cancer.

## INTRODUCTION

Colorectal cancer (CRC), the third most common type of cancer in the world, is one of the leading causes of cancer deaths with a worldwide incidence of more than one million cases per year [1–5]. In addition, the incidence rates of colorectal cancer have continued to increase in both the sexes from 1999 to 2010 [4]. CRC develops and progresses over several years with distinct molecular and cytological characteristics, eventually resulting in a carcinoma with a high rate of invasion and metastasis [3, 6]. Metastasis of CRC to the lymph nodes is the most prevalent, followed by metastasis to the liver, influencing approximately half of the CRC patients with the 5-year survival rate of 11.7% after the diagnosis [7–9].

Despite recent advances in the screening, surgical resection, systemic chemotherapies, and treatment modalities of CRC, which led to a higher rate of response and better quality of life, most of the key signaling pathways involved in its progression still need to be identified and characterized. It is suggested that the most important factors contributing to progression and poor prognosis of the cancer are recurrence and metastasis [10–12]. There have been multiple reports suggesting that epithelial-mesenchymal transition (EMT) is one of the key events in tumor metastasis. In addition, EMT is known to be implicated in many important events such as invasion, tumor progression, resistance to chemotherapy and the acquisition of stem cell-like characteristics [13–20]. EMT is a reversible process, during which, epithelial cells exhibit some properties of mesenchymal cells such as proteolysis and motility while losing many of their epithelial characteristics such as cell-cell adhesion and cell polarity [21]. The major molecular players involved in EMT are the Wnt signaling pathway, tyrosine/serine/threonine receptor kinases, and transcriptional regulators including SNAI, ZEB, and TWIST [13, 15–17, 21]. The cross talk of these signaling pathways and their novel components requires to be characterized to understand the role of EMT in tumor progression.

Interferon-induced transmembrane protein 1 (IFITM1) is a member of the IFN-inducible transmembrane protein family, and is also known as 9-27 or CD225. It is initially identified as Leu 13 and a component of membrane complex that is implicated in proliferation, homotypic adhesion in lymphocytes, and metastasis [22–27]. IFITM1 is involved in host-pathogen interaction, and IFN-α and IFN-γ were found to induce the expression of IFITM1 in response to pathogens [25]. Recently, IFITM1 was shown to be highly expressed in several cancers, including cervical, esophageal, ovarian, brain, and colon cancer [27–31]. Functionally, the importance of IFITM1 was reported in glioma cell lines and head and neck squamous cell carcinoma (HNSCC) where IFITM1-depleted cancer cells displayed a lower level of proliferation and invasion [33, 34]. It was shown that IFITM1 is related to Wnt signaling as IFITM1 is highly induced by the activation of the b-catenin pathway [32]. However, the functional significance and signaling mechanism of IFITM1 in the progression of various cancers remain unanswered. Here, we attempted to study the critical role of IFITM1 in colorectal cancer development and progression using patient-derived samples as well as several colorectal cancer cell lines. We report that IFITM1 is essential for the migration and invasion of colorectal cancer and is also associated with a poor prognosis of the disease.

## RESULTS

### IFITM1 is highly expressed in metastatic colorectal cancer cell lines

**Figure 1:**
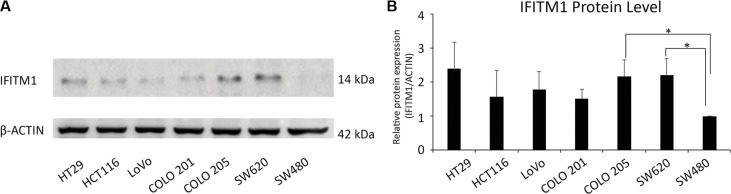
Evaluation of IFITM1 expression in various colorectal carcinoma cell lines by western blot (**A**) IFITM1 expression was evaluated in colorectal cancer cell lines. Cell lysates were obtained from the indicated cancer cell lines, and immunoblots were performed to evaluate the expression of IFITM1. ACTIN was used as the loading control. (**B**) Proteins were quantified by Image J and expressed as the ratio of IFITM1 to ACTIN (*n* = 3, *p < 0.05).

As IFITM1 has been shown to be increased in cervical, esophageal, ovarian, brain and colon cancers [27–31], we analyzed the expression of IFITM1 in multiple patient-derived colorectal cancer cell lines by immunoblot. SW480, a non-metastatic cell line, exhibited a low level of IFITM1 expression, whereas metastatic cancer cell lines such as HT29, HCT116, and SW620 exhibited a high level of IFITM1 expression (Figure 1A and 1B), suggesting that IFITM1 might be linked to metastasis and progression of colorectal cancer.

### shRNA-mediated knockdown of IFTM1

**Figure 2:**
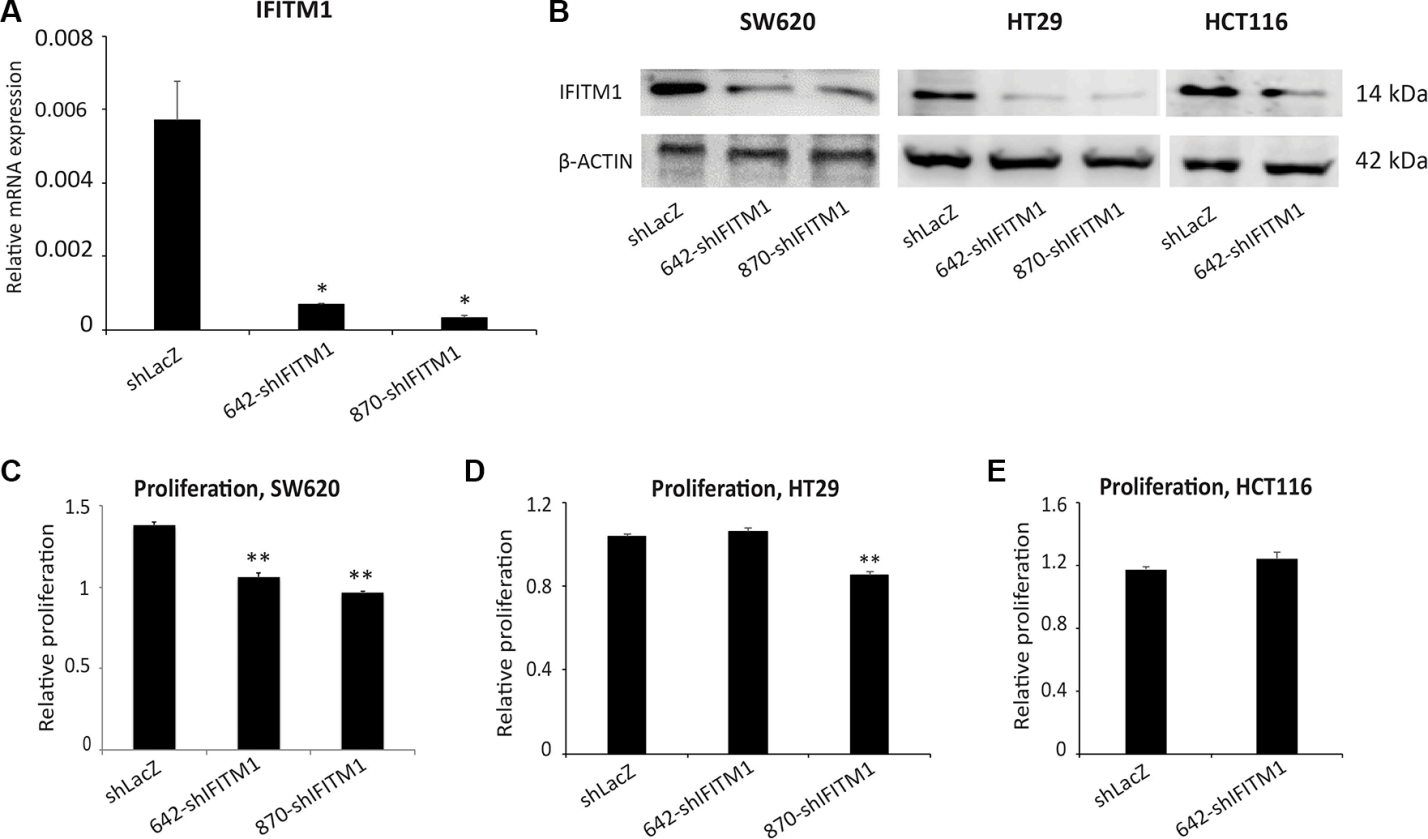
IFITM1 depletion modestly impairs cell proliferation in colorectal cancer cell lines Colorectal cancer cell lines were infected with either control (shLacZ) virus or IFITM1 knockdown virus (642-shIFITM1 and 870-shIFITM1) and selected with puromycin. RNA and proteins were isolated to determine the expression of IFITM1. (**A**) RT-qPCR was performed to determine IFITM1 mRNA expression in SW620 cells after infection. (*p < 0.05). (**B**) Immunoblot with anti-IFITM1 antibody was conducted to analyze IFITM1 protein level, and ACTIN was used as the loading control. (**C**–**E**) Control (shLacZ) or IFITM1-depleted (shIFITM1) colorectal cancer cell lines were incubated for 72 hrs to determine the proliferation rate by MTT assay. Data shown are from two to three experiments (*p < 0.05, **p < 0.001).

To determine the functional importance of IFITM1 in colorectal cancer, we used shRNA to inhibit the expression of IFITM1 in cancer cell lines. Colorectal cancer cell lines were transduced with either nonsense control (shLacZ) or IFITM1 shRNA (shIFITM1) and selected with puromycin. To minimize off-target effects, two independent shRNAs, 642-shIFITM1 and 870-shIFITM1, were used in this study. RNA and cell lysates from transduced cells were extracted to determine the expression level of IFITM1. The mRNA level of *IFITM1* was significantly decreased by about 90% in IFITM1 shRNA-transduced cells compared to nonsense control (Figure 2A). As mRNA expression does not necessarily correlate with the protein level, we determined the protein levels of IFITM1 after shRNA transduction. Cell lysates were obtained from shRNA-transduced cells and immunoblots were performed using anti-IFITM1 antibody (Figure 2B). IFITM1 protein level was also decreased in IFITM1 shRNA-transduced colorectal cancer cell lines (SW620, HT29 and HCT116), compared to that in the control cells. These data led us to confirm that the expression of IFITM1 was successfully depleted at both RNA and protein levels.

### The proliferation of colorectal cancer cell lines is modestly impaired by the depletion of IFITM1

To test whether IFITM1 was involved in the growth of colorectal cancer cells, we determined the proliferation ability using MTT assay after IFITM1 knockdown. Control or IFITM1 shRNA-transduced cancer cells including SW620, HCT116 and HT29 were incubated for 72 hours to determine proliferation *in vitro*. The proliferation rate of IFITM1-depleted SW620 and HT29 was decreased by about 10∼35% of control cells, suggesting that the proliferation was modestly impaired in the absence of IFITM1 in colorectal cancer cell lines including SW620 and HT29, but not in HCT116 (Figure 2C–2E).

### IFITM1 is essential for the mobility of colorectal cancer cells

**Figure 3:**
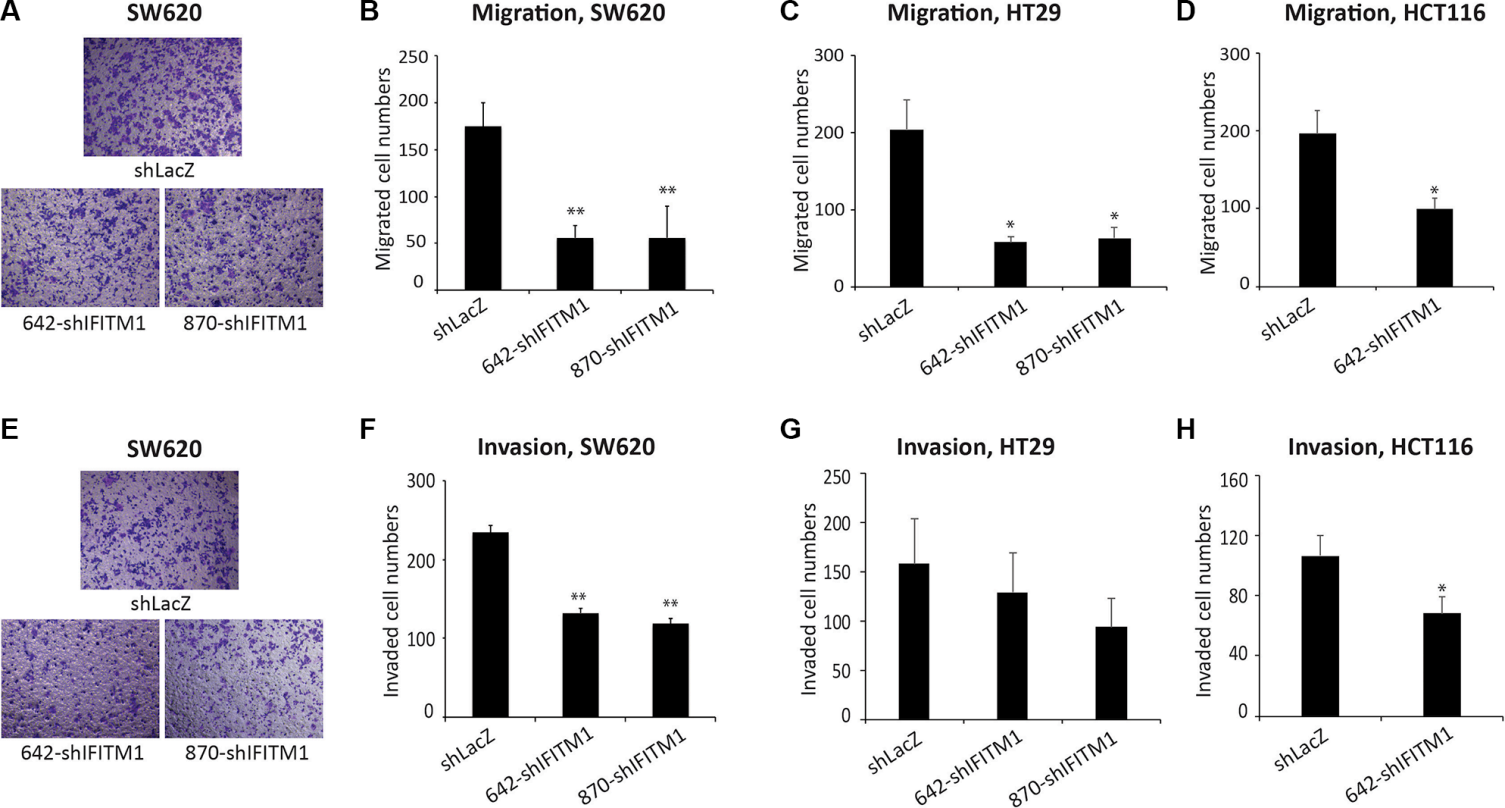
IFITM1 depletion impairs migration and invasion of colorectal cancer cell lines *in vitro* Control (shLacZ) or IFITM1-depleted (shIFITM1) colorectal cancer cell lines were seeded in a matrigel-uncoated and coated transwell, followed by incubation for 18 hrs and 24 hrs for assessing migration and invasion, respectively. Cells that had migrated to the lower surface of the transwell were stained and quantified. (**A** and **E**) Imaging was done using an inverted microscope (magnification: 100×) and representative images are shown. Data shown (A–D: migration; **E**–**H**: invasion) are from three experiments (*p < 0.05, **p < 0.001).

As IFITM1 promotes the invasion of glioma cell lines and head and neck squamous cell carcinoma (HNSCC) [33, 34], we examined whether IFITM1 displays a similar phenotype in colorectal cancer. Cancer cell lines (SW620, HT29, and HCT116) transduced with either shLacZ or IFITM1 shRNA were seeded on an insert of the transwell system and incubated for 18 hrs to examine migration. The cells on the inserts of transwell system were stained and counted to evaluate the migratory ability of cancer cells. IFITM1 shRNA-transduced cells migrated about 25∼40% of control-transduced cells in the cell lines tested (Figure 3A–3D). We also determined whether IFITM1 was required for the invasion of cancer cells *in vitro*. Control or IFITM1-depleted cells were seeded on a matrigel-coated insert of transwell system and incubated for 24 hrs to assess invasion ability. As shown in Figure 3E–3H, IFITM1 shRNA-transduced cells exhibited about a half of the invasion ability of control-transduced cells. These results suggest that IFITM1 is essential for the migration and invasion of colorectal cancer cells, thus possibly contributing to the poor prognosis of colorectal cancer.

### IFITM1 is required for the expression of epithelial mesenchymal transition (EMT) signature

**Figure 4:**
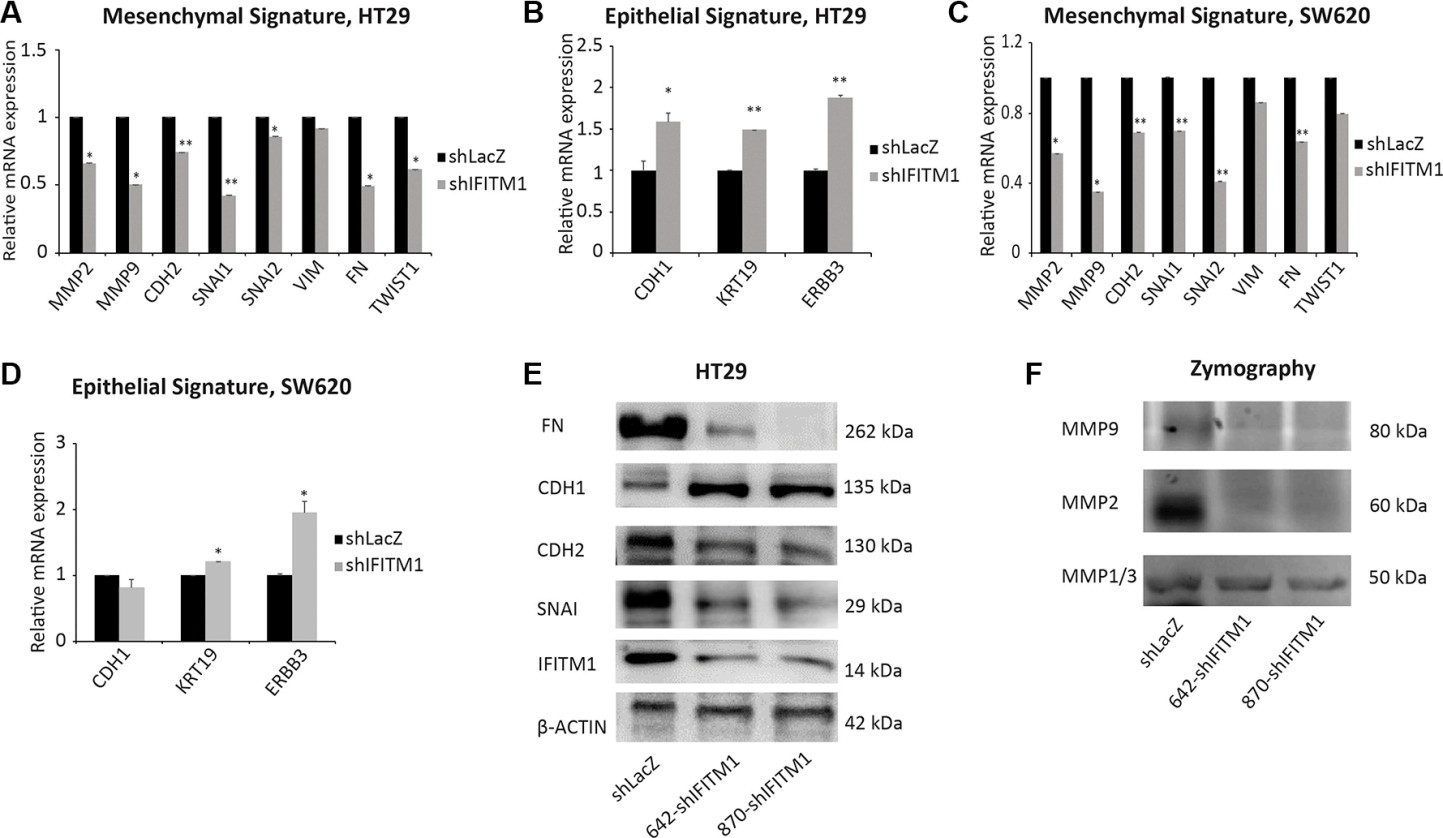
IFITM1 is required for the maintenance of EMT signature (**A**–**D**) RNA was isolated from control (shLacZ) or IFITM1-depleted (shIFITM1) cancer cell lines (A and B: HT29; C and D: SW620) and EMT signature genes were determined by RT-qPCR (*p < 0.05, **p < 0.001). (**E**) Cell lysates were obtained from control (shLacZ) or IFITM1-depleted (shIFITM1) HT29 cells and immunoblots were conducted with antibodies indicated. ACTIN was used as a loading control. (**F**) Supernatants obtained from control (shLacZ) or IFITM1-depleted (shIFITM1) HT29 cells were analyzed for enzymatic activity of MMP1/3, MMP2 and MMP9.

As EMT has been implicated in the migration and invasion of cancer cells [21, 35], we examined whether the migratory defect of colorectal cancer cells in the absence of IFITM1 was related to the expression of EMT signature. Total RNA was isolated from control cells or IFITM1-depleted colorectal cancer cell lines, and RT-qPCR was performed to analyze the expression of several genes associated with epithelial and mesenchymal characteristics. The genes maintaining mesenchymal properties such as *MMP2*, *CDH2*, *MMP9*, *SNAI1*, *SNAI2* and *FN* were significantly decreased in the absence of IFITM1 whereas epithelial-related genes such as *ERBB3* and *KRT19* were increased (Figure 4A–4D). Then, we analyzed EMT signature at protein levels by western blot as well. In consistent with the RNA level, protein levels of FN, CDH2 and SNAI1 were much lower in the absence of IFITM1 (Figure 4E). MMPs are digestive enzymes that degrade the basement membrane and implicated in invasion [36]. Consistent with the mRNA expression of *MMP2*, gelatin zymography assay revealed that the enzymatic activity of MMP2 was significantly lower in the absence of IFITM1 (Figure 4F). These results suggest that IFITM1 is required to maintain EMT signature in colorectal cancer.

### IFITM1-mediated EMT signature is associated with Caveolin-1

**Figure 5:**
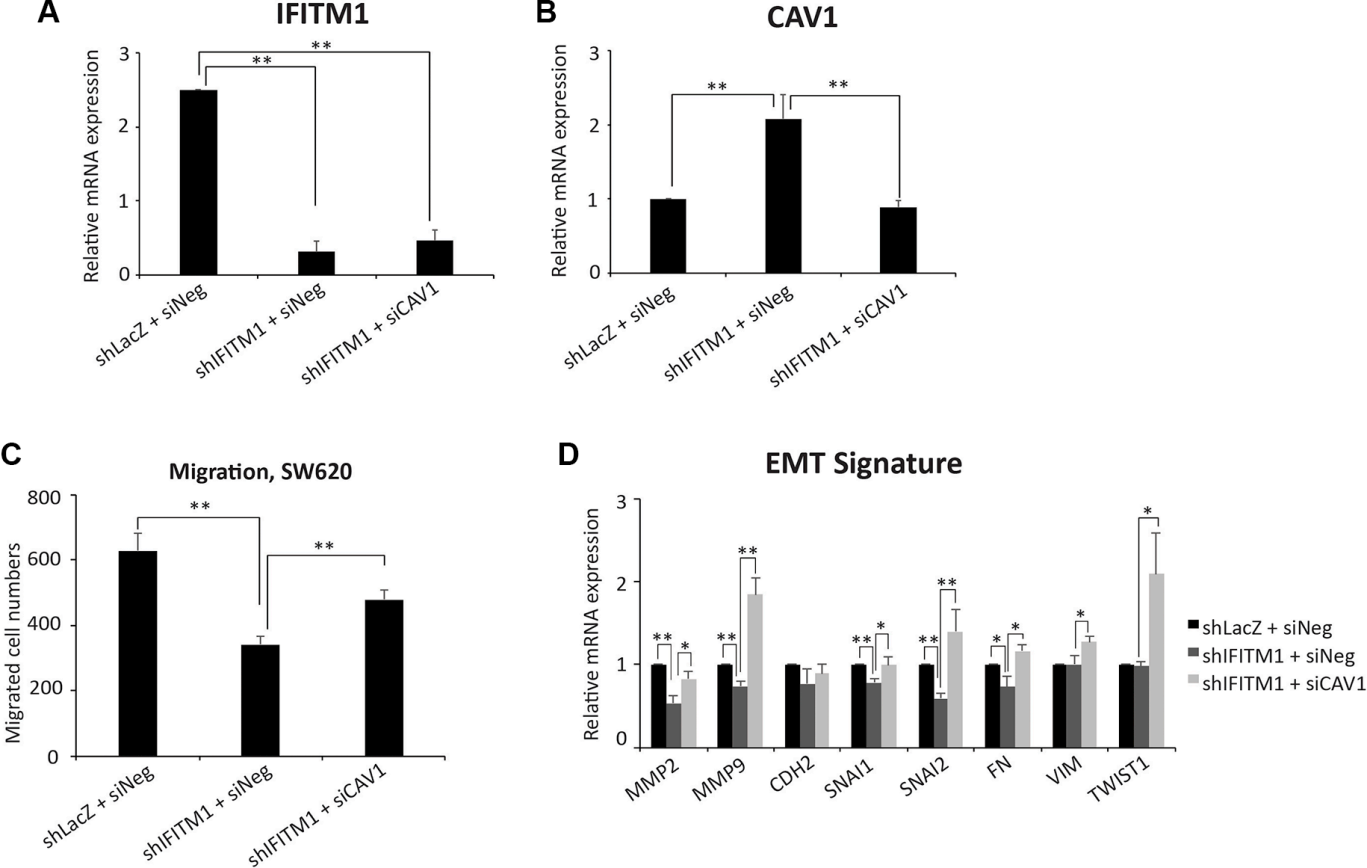
IFITM1 mediates EMT signature through Caveolin-1 Control (shLacZ) or IFITM1-depleted (shIFITM1) SW620 cells were treated with either control siRNA or siCAV1 for 2 days and RT-qPCR was conducted to measure the mRNA expression of IFITM1 (**A**) Caveolin-1 (**B**) and EMT signature (**D**) (*p < 0.05, **p < 0.001). (**C**) Control (shLacZ) or IFITM1-depleted (shIFITM1) SW620 cells were treated with either control siRNA or siCAV1 and then analyzed for migration ability (C). Data shown are from three experiments (**p < 0.001).

It has been identified recently that Caveolin-1 (CAV1) is a downstream target of IFITM1 [27]. Thus, we determined whether IFITM1-mediated EMT signature is mediated by CAV1 in SW620. Control or IFITM1-knockdowned cells were transiently transfected with either negative control (siNeg) or siCaveolin-1 (siCAV1), and the expression of IFITM1 and CAV1 is determined by RT-qPCR. CAV1 expression is significantly increased following knocking IFITM1 down, which was in turn reduced by siCAV1 transfection (Figure 5A and 5B). Thus, we determined whether the impaired migratory ability in the absence of IFITM1 is mediated by CAV1. The defective migratory ability of SW620 with the loss of IFITM1 was partially rescued by the inhibition of Caveolin-1 (Figure 5C). Further, the EMT signature such as *MMP2*, *MMP9*, *SNAI1*, *SNAI2*, and *FN* that was decreased in the absence of of IFITM1 was partially rescued by CAV1 inhibition (Figure 5D). These results suggest that the change of EMT signature by IFITM1 is mediated through Caveolin-1.

### Ectopic expression of IFITM1 promotes the migratory capacity of colorectal cancer cells

**Figure 6:**
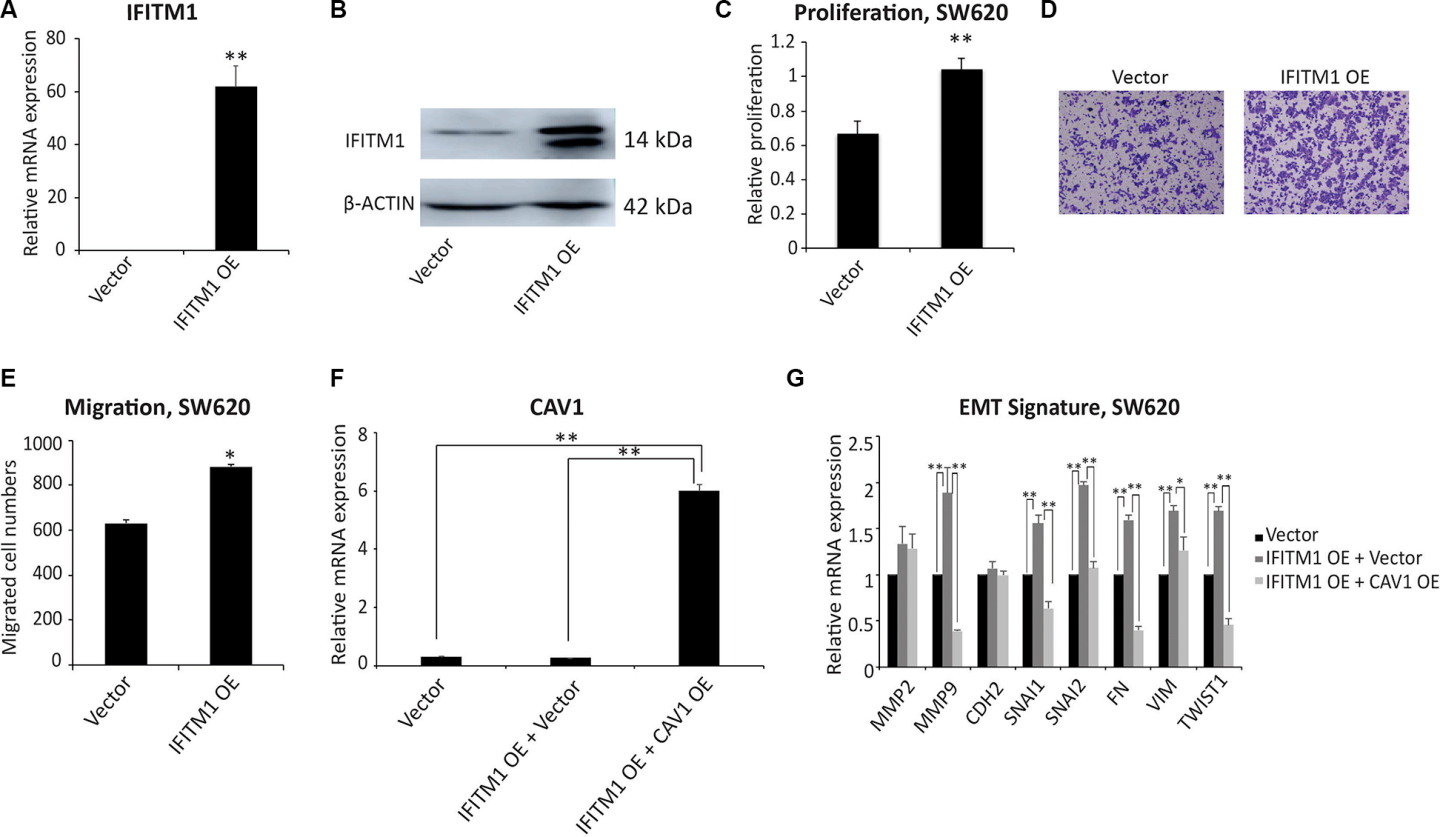
Ectopic expression of IFITM1 promotes the proliferation and migration of SW620 cancer cells *in vitro* (**A** and **B**) SW620 cells were transiently transfected with either control (vector) or IFITM1 expression construct (IFITM1 OE). RNA and proteins were isolated to examine the expression of IFITM1. (A) RT-qPCR was performed to determine IFITM1 mRNA expression in SW620 cells after transfection (**p < 0.001). (B) IFITM1 protein level was analyzed by immunoblot using anti-IFITM1 antibody, and ACTIN was used as the loading control. (**C**) SW620 cells transfected with control (vector) or IFITM1-expression construct (IFITM1 OE) were incubated for 72 hrs to determine the proliferation by MTT assay. Data shown are from three experiments (**p < 0.001). (**D** and **E**) SW620 cells transfected with control (vector) or IFITM1-expression construct (IFITM1 OE) were seeded in a transwell for assessing migration. Cells were incubated for 18 hrs, stained, and quantified. (D) Representative images are shown. (**E**) Data shown are from three experiments (*p < 0.05). (**F** and **G**) SW620 cells were transiently transfected with either Vector alone, IFITM1-overexpressing (IFITM1 OE) and Vector, or IFITM1 OE and CAV1-overexpressing (CAV1 OE) constructs. RNA was isolated from the transduced cells, and CAV1 (F) and EMT signature (G) were quantified by RT-qPCR (*p < 0.05, **p < 0.001).

To understand whether IFITM1 is sufficient to drive the migration of cancer cells, we performed a gain of function of IFITM1 in colorectal cancer cells. Here, we ectopically expressed IFITM1 in cancer cells to examine whether IFITM1 could promote proliferation and migration of cancer cells. SW620 cancer cells were transiently transfected with either control vector or IFITM1 expression construct (IFITM1 OE) and IFITM1 expression was confirmed by RT-qPCR and immunoblot. As shown in Figure 6A and 6B, IFITM1 was highly expressed at both mRNA and protein levels. In line with the results obtained by shRNA-mediated knockdown (Figures 2, 3, 4), ectopic expression of IFITM1 increased the proliferation as well as migration ability of colon cancer cells (Figure 6C–6E). Furthermore, the expression of EMT genes was dysregulated with overexpression of IFITM1 (Figure 6F). Specifically, ectopic IFITM1 expression resulted in a significant increase in the expression of mesenchymal-related genes such as *MMP9*, *SNAI1*, *SNAI2*, *FN*, *VIMENTIN*, and *TWIST1* (Figure 6G). Also, these genes were in turn decreased by coexpression of CAV-1 and IFITM1 (Figure 6F and 6G). Collectively, these results suggest that IFITM1 is essential for the migration of colorectal cancer cells and is implicated in maintaining EMT signature through CAV1.

### IFITM1 is associated with a poor prognosis of colorectal cancer patients

**Figure 7:**
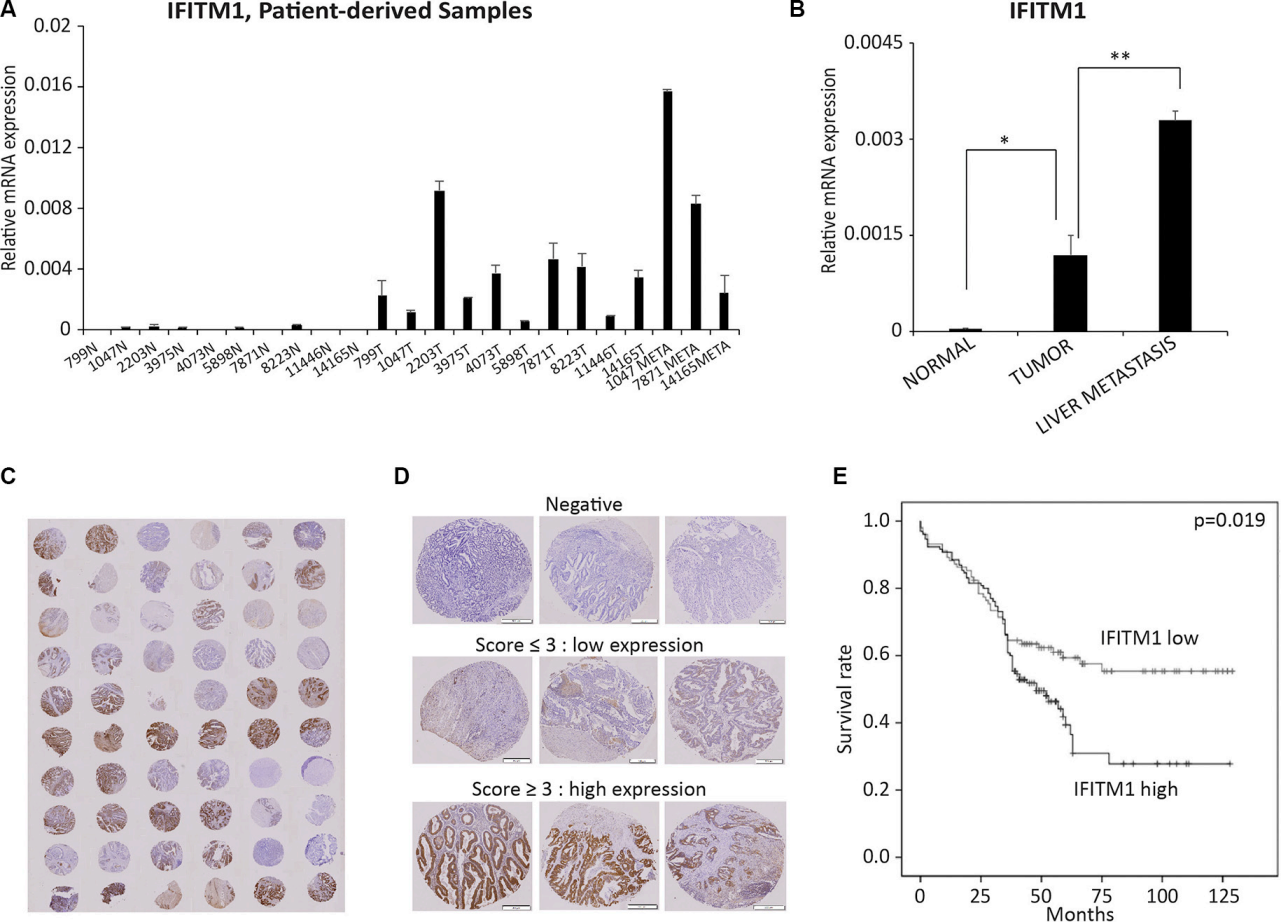
IFITM1 expression is associated with poor prognosis of colorectal cancer patients (**A** and **B**) RNA was isolated from formalin-fixed and paraffin-embedded (FFPE) tissues and *IFITM1* was quantified by RT-qPCR. (N: normal; T: tumor; META: metastasis samples). Individual samples (A) and average of each group of samples (B) are shown (*p < 0.05, **p < 0.001). (**C**–**E**) 232 colorectal cancer samples were stained with IFITM1 antibody and graded based on both staining intensity and staining frequency. (C and D) Representative images of tissue microarray and immunohistochemistry are shown. (E) Survival rate was determined based on the expression of IFITM1 expression using Kaplan-Meier method (*p* < 0.05).

To determine the biological relevance of the results obtained from colorectal cancer cell lines, we analyzed the expression of IFITM1 and its association with patient survival rate using colorectal cancer patient-derived samples. We analyzed *IFITM1* mRNA level in 23 patient-derived samples obtained from normal area (*n* = 10), tumor area (*n* = 10) and metastatic area (*n* = 3) by RT-qPCR. IFITM1 was almost undetectable in samples from normal area, whereas it was highly increased in samples from tumor and metastatic regions (Figure 7A and 7B). These results indicate that increased IFITM1 expression is associated with progression of colorectal cancer. Tissue microarray (TMA) block sections obtained from 232 patients with colorectal cancer were stained with IFITM1 antibody to correlate IFITM1 expression with survival rate and hazard ratio (Figure 7C–7E and Table 1). Based on both the intensity of IFITM1 staining and the frequency of IFITM1-positive cells, samples were sub-divided into two groups, IFITM1 low and IFITM1 high, and the association of IFITM1 expression with patient prognosis was determined (Figure 7C–7E). As shown in Figure 7E, IFITM1 expression was significantly associated with patient survival rate. Specifically, 28% of the patients expressing high levels of IFITM1 survived after surgery, whereas approximately 55% of the patients expressing low levels of IFITM1 survived. These results suggest that IFITM1 is a prognostic marker in colorectal cancer patients, and an attractive therapeutic target to block the progression of colorectal cancer.

**Table 1:** Association of IFITM1 expression with clinicopathological factors by logistic regression analysis

Clinicopathologic factors	Variable	Univariate analysis		Multivariate Analysis	
		HR (95%/CI)	*P* value	HR (95%/CI)	*P* value
Age	<60 yr vs. ≥60 yr	1.516 (1.008–0.280)	0.046	1.595 (1.030–2.471)	0.036
Sex (0 = M, 1 = F)	Female vs. Male	0.732 (0.508–1.057)	0.096	0.742 (0.057–1.086)	0.125
pT stage			**0.000**		0.056
1		1.000		1.000	
2		2.486 (0.724–8.535)		1.857 (0.499–6.907)	
3		2.164 (0.775–7.832)		1.251 (0.260–6.027)	
4		3.008 (1.823–19.794)		2.588 (0.492–13.614)	
pN stage			**0.000**		0.079
0		1.000		1.000	
1		1.459 (0.947–2.248)		10.386 (1.132–95.325)	
2		3.066 (1.941–4.843)		12.773 (1.362–119.787)	
Vascular invasion	Negative vs. Positive	2.395 (1.561–3.675)	**0.000**	1.334 (0.744–2.392)	0.333
Lymphatic invasion	Negative vs. Positive	2.291 (1.567–3.351)	**0.000**	1.407 (0.822–2.409)	0.213
Stage			**0.001**		**0.031**
I		1.000		1.000	
II		1.410 (0.738–2.692)		1.483 (0.485–4.533)	
III		2.225 (1.192–4.152)		0.174 (0.016–1.912)	
IV		4.763 (1.940–11.691)		0.600 (0.063–5.731)	
IFITM1 expression	Low vs. High	1.574 (1.071–2.314)	**0.021**	1.628 (1.095–2.420)	**0.016**

## DISCUSSION

In this study, we find that IFITM1 is essential in migration/invasion of colorectal cancer and is required for the expression of EMT signature in the disease. In addition, it is a plausible biomarker to predict the progression of the disease. These findings are consistent with recent reports, where it was shown that IFITM1 is associated with proliferation or invasion/metastasis in several cancers, including glioma, colorectal cancer, and head and neck cancer [27, 33, 34]. Andreu et al. also reported that this molecule is highly increased by the activation of Wnt signaling [32]. In esophageal squamous cell carcinoma (ESCC), IFITM1-depleted cancer cells displayed a high sensitivity to cisplatin treatment [37]. However, the data obtained with chronic myeloid leukemia (CML) are quite different from those obtained with the solid cancers described above. CML patients expressing higher levels of IFITM1 displayed a better survival rate compared to patients with lower levels of IFITM1 [38]. Therefore, the variable nature of association of IFITM1 with cancer prognosis suggests that it should be carefully approached to utilize IFITM1 as a biomarker.

In colorectal cancer, metastatic spread is one of the crucial factors linked to a high level of mortality. However, the signaling pathways involved in metastasis are not completely understood, and thus identifying novel pathways in metastasis may contribute to higher survival rates of cancer patients [39, 40]. EMT, an important process implicated in various aspects of tumor progression, such as invasion, metastasis and drug resistance, is currently being investigated actively [13–20]. Our data show that the EMT signature is influenced by IFITM1 and CAV1; however, the mechanism by which IFITM1/CAV1 control EMT signature remains unexplored. It has been shown that reduction of CAV1 increased the transcriptional activation of b-catenin, a key transcription factor of Wnt signaling pathway, resulting in enhanced tumor invasion [41]. Thus, we propose that the change of EMT signature by IFITM1 might be mediated indirectly by CAV1-induced change of transcription factors such as b-catenin. To prove this possibility, which is beyond the scope of current study, several strategies such as CHIP-seq, immunoprecipitation, RNA-seq, etc. may be required. Nevertheless, our findings position IFITM1 as an important signaling mediator in EMT, which contributes to the progression of colorectal cancer. Furthermore, the fact that IFITM1 is a cell surface molecule makes it an attractive target for antibody-mediated therapy. Thus, it is suggested that developing small molecules or antibodies to block the function of IFITM1 could pave an effective way to prevent the disease from developing and progressing into advanced stages of the disease.

## MATERIALS AND METHODS

### Cell lines

Human colorectal cancer cell lines HCT116, HT29, LoVo, SW480, SW620, COLO201, and COLO205 were purchased from the Korean Cell Line Bank (KCLB). HCT116 cells were grown in DMEM medium (Corning, USA), and HT29, LoVo, SW480, SW620, COLO201, COLO205 and LoVo cells were grown in RPMI-1640 medium (Corning, USA) supplemented with 10% Fetal Bovine Serum (Corning, USA), 1% MEM essential amino acids (Corning, USA) and 1% penicillin/streptomycin (Gibco, USA) at 37°C in a humidified atmosphere containing 5% CO_2_.

### Lentiviral constructs/siRNA and transfection

Short hairpin RNA (shRNA) constructs were designed and cloned into the pLV-RNAi lentiviral backbone as per the manufacturer’s protocol (Biosettia, USA). The target sequences for 870-shIFITM1 and 642-shIFITM1 are 5’-TGTCTACAGTGTCATTCAATA-3’ and 5’-TGTGACAGTCTACCATATTA-3’, respectively. The sequence of nonsense shRNA was provided by Biosettia. Virus was produced in 293T cells transfected with viral constructs along with psPAX2 and pMD2 constructs. Viral supernatants were collected on day 2 and 3 after transfection and used to infect target cells. The open reading frame (ORF) of IFITM1 (Forward, 5′-GGC GCATATCTCGAGATGCACAAGGAGGAACATGAGG TG-3′ and reverse, 5′-GGCGCATATGAATTCGGTAACC CCGTTTTTCCTGTATTATCTGTA-3′) and Caveolin-1 (CAV1) (Forward, 5′-ACCGCTCGAGATGTCTGGGG GCAAATACGTAGAC-3′ and Reverse, 5′-ACGCGGAT CGGCTATTTCTTTCTGCAAGTTGATGCG-3′) were amplified and cloned into pLVX-AcGFP-N1 (Clontech). Negative siRNA duplex (sense, 5′-UCCUCCGAACGU GUCACGUTT-3′, antisense, 5′-ACGUGACACGUUCG GAGAATT-3′) or siCAV1 (sense, 5′-AACCAGAAGGGA GACACAG-3′, antisense, 5′-CUGUGUGUCCCUUCU GGUU-3′) (GenePharma) were transfected using X-tremeGENE siRNA Transfection Reagent according to the manufacturer’s instructions (Roche, Germany).

### RNA extraction and realtime qPCR

RNA was isolated using Trizol (Invitrogen, USA) or Hybrid R (Gene All, Korea), and converted to cDNA using ReverTra Ace^®^ qPCR Kit (TOYOBO, Japan) according to the manufacturer’s instructions. To determine the level of gene expression, RT-qPCR was performed using the qPCR Master Mix Kit (TOYOBO, Japan). Primer sequences for RT-qPCR are shown in Supplementary Table S1.

### Western blotting analysis

Cell lysates were harvested using RIPA lysis buffer for 30 mins on ice and centrifuged at 13,000 rpm for 10 mins at 4°C. Protein concentration of the supernatant was determined by Bio-Rad Protein Assay (Bio-Rad Laboratories, Inc., USA). An equal amount of each protein extract (30 μg) was resolved using 10% polyacrylamide gel and electro-transferred onto 0.45 μm hybridization nitrocellulose filter (HATF) membrane (Millipore, USA) using Trans-blot Turbo (Bio-Rad Laboratories, Inc., USA). Membranes were immunoblotted with either rabbit polyclonal anti-IFITM1 antibody (GeneTex, USA), rabbit polyclonal anti-actin antibody (Abcam, USA), rabbit monoclonal anti-SNAIL antibody (Cell Signaling, USA), rabbit monoclonal anti-E-Cadherin antibody (Cell Signaling, USA), mouse monoclonal anti-Fibronectin antibody (Abcam, USA), or mouse monoclonal anti-N-Cadherine antibody (BD, USA) overnight at 4°C. Membranes were incubated with either HRP-conjugated anti-rabbit immunoglobulin (Cell Signaling, USA) or HRP-linked anti-mouse immunoglobulin (Cell Signaling, USA) for 1 hr at room temperature. The protein signal was detected by enhanced chemiluminescence (Thermo, USA) using the Amersham Imager 600 (GE Healthcare Life Sciences, UK).

### Cell proliferation assay (MTT assay)

MTT assay was used to evaluate cell proliferation by using Cell Proliferation Kit I according to the manufacturer’s instructions (Roche, Germany). Briefly, cells (5 × 10^3^) were seeded into a 96-well plate and incubated for an additional 96 hrs. Cells were incubated in 5 mg/ml of MTT solution for 4 hrs and then solubilized with 100 μl solubilization solution (10% SDS in 0.01 M HCl) overnight. Absorbance was read at 575 nm and 650 nm using a plate reader.

### Migration and invasion assay

Cell migration and invasion were analyzed *in vitro* using the transwell insert system (Corning, USA) without coating or with coating by 20 μL of Matrigel (BD, USA), respectively. The culture insert was attached on bottom of a 24-well plate, and 100 μL of serum-free media containing 1.5 × 10^5^ cells were seeded into each well of the insert. Six-hundred μL of media containing 10% FBS was added outside the transwell culture insert. Cells were incubated at 37°C for 18 hrs and 24 hrs in a humidified atmosphere with 5% CO_2_ for migration and invasion, respectively. Transwells were washed twice with PBS and cleaned using cotton swap. The cells were fixed with 1% formaldehyde for 15 mins, washed twice with PBS, stained with 0.1% of crystal violet for 15 mins and then observed using a microscope (Leica, Germany).

### Gelatin zymography analysis

Cells were incubated in the media without serum for 24 hrs and the supernatant was collected to determine the activity of MMPs. Samples were analyzed on SDS-PAGE containing 0.1% gelatin. After electrophoresis, the gel was renatured two times with 2.5% Triton X-100 for 30 mins at room temperature, followed by washing with ddH_2_O. The gel was incubated in developing buffer (50mM Tris-HCl pH 7.6, 50 mM NaCl, 10 mM CaCl_2_, 0.05% Brij 35) for 24 hrs at 37°C. Gel staining was conducted for 1 hr at room temperature using Coomassie Brilliant Blue Protein Staining and destained using destaining solution (methanol : ddH_2_O : acetic acid = 5 : 4 : 1) at room temperature.

### Human colorectal carcinoma specimens

A total of 232 colorectal carcinoma tissue specimens were obtained from Soonchunhyang University Cheonan Hospital, Korea, where samples were collected from patients who underwent surgery between 2002 and 2006. These tissues were formalin-fixed and paraffin-embedded (FFPE). Clinicopathological data including age, gender, TNM classification, invasion of blood vessel and lymphatic vessel were shown at Table 2. Tumor stage was identified according to the American Joint Committee on Cancer’s TNM classification system. Sample collection for this study was approved by the Ethics Committee of Soonchunhyang University, Cheonan Hospital (NON2014-006).

**Table 2:** Clinicopathological features of patient samples

Clinicopathological factors	*N*
Age, years, mean (SD)	63.1 (± 12.6)
Gender, *N* (%)	
F	98 (42.2)
M	134 (57.8)
pT stage, *N* (%)	
1	14 (6.0)
2	34 (14.7)
3	146 (62.9)
4	38 (16.4)
pN stage, *N* (%)	
0	130 (56.0)
1	64 (27.6)
2	38 (16.4)
Vascular invasion, *N* (%)	
0	195 (84.1)
1	37 (15.9)
Lymphatic invasion, *N*(%)	
0	170 (73.3)
1	62 (26.7)
Sage, *N* (%)	
I	36 (15.5)
II	91 (39.2)
III	95 (40.9)
IV	10 (4.3)

Total of 232 cases of colorectal cancer patient samples were used in this study. Tumor stage was classified according to the American Joint Committee on Cancer's tumor-node-metastasis (TNM) classification.

### Tissue microarray (TMA) and immunohistochemistry (IHC) assay

Immunohistochemical staining was performed using tissue microarray (TMA) block sections to determine IFITM1 expression in colorectal cancer patient samples. Each TMA block contained 60 cores (2 mm of size) from 30 samples. For immunohistochemistry, 4-μm sections were obtained using a microtome, deparaffinized in xylene, and rehydrated in 100% to 70% alcohol series. Antigen retrieval was achieved in citrate buffer (pH 6.0) using a microwave for 15 mins. To eliminate endogenous peroxidase activity, the sections were incubated in peroxidase blocking solution (Dako, Denmark) for 30 mins and then washed with phosphate-buffered saline containing 0.1% Tween 20 (PBST). The sections were incubated with anti-rabbit IFITM1 antibody (GeneTex, 1:500) for 2 hrs at room temperature, followed by incubation in enhancer for 30 mins and treatment with polymer for 1 hr at room temperature. After washing with PBST, sections were incubated with DAB, counterstained with hematoxylin, and observed under a microscope.

### IHC data analysis

The IFITM1-stained tissue cores were examined by 2 independent observers (CJK and DJJ), and a consensus score was determined for each specimen. A positive reaction was scored into 4 grades, according to the intensity of the staining: 0, 1+, 2+, and 3+. The percentages of IFITM1-positive cells were also scored into 4 categories: 0 (0%), 1 (1–30%), 2 (31–70%), and 3 (71–100%). The final score, calculated as the product of the intensity score multiplied by the percentage score, was classified as follows: 0 for negative; 1–3 for weak; 4–6 for moderate; and 7–9 for strong. Samples with a final score ≤ 3 were grouped together as IFITM1 expression negative while those with a score ≥ 4 were grouped together as IFITM1 expression positive.

### Statistical analysis

Statistical analysis was conducted using SPSS 19.0 (Chicago, IL, USA) program. The results of RT-qPCR, migration and invasion were analyzed with Student’s *T-test*. Hazard ratio and 95% confidence interval of clinicopathological data were evaluated using Cox regression models. Kaplan-Meier method was used to analyze disease-free survival rate using the log-rank test. A *p-value* of less than 0.05 was considered statistically significant in all assessments.

## SUPPLEMENTARY TABLE


