# Supplementary material for: Interferon-induced transmembrane protein 1 (IFITM1) is required for the progression of colorectal cancer
Source: Oncotarget. 2016 Dec 11;7(52):86039–50. doi: 10.18632/oncotarget.13325 (PMC5349895; doi:10.18632/oncotarget.13325)
Supplement: Supplementary file 1 [file oncotarget-07-86039-s001.pdf]

Interferon-induced transmembrane protein 1 (IFITM1) is required for the progression of colorectal cancer

Supplementary Materials

Supplementary Table 1: Primers used for realtime qPCR

No.	Target Gene	Sequence (5′–3′)
1.	IFITM1-F	CATCCGGAAGAAACTGGT
	IFITM1-R	TCCCACAAAGCCAACTC
2.	B2M-F	TGAAGCTGACAGCATTCGG
	B2M-R	CTGCTGGATGACGTGAGTAAA
3.	MMP2-F	CGCTCAGATCCGTGGTGA
	MMP2-R	CGCCAAATGAACCGGTCCTT
4.	MMP9-F	TTGACAGCGACAAGAAGTGG
	MMP9-R	GCCATTCACGTCGTCCTTAT
5.	TIMP1-F	AGACCTACACTGTTGGCTGTGAG
	TIMP1-R	GACTTGAAGCCCTTTTCAGAG
6.	TIMP2-F	ATGCACATCACCCTCTGTGA
	TIMP2-R	CTCTGTGACCCAGTCCATCC
7.	P27-F	TGCAACCGACGATTCTTCTACTCAA
	P27-R	CAAGCAGTGATGTATCTGATAAACAAGGA
8.	CYCLIN D1-F	ACAAACAGATCATCCGCAAACAC
	CYCLIN D1-R	TGTTTGGGCTCCTCAGGTTC
9.	CDK2-F	GCTAGCAGACTTTGGACTAGCCAG
	CDK2-R	AGCTCGGTACCACAGGGTCA
10.	E-Cad-F	TTGCACCGGTCGACAAAGGAC
	E-Cad-R	TGGATTCCAGAAACGGAGGCC
11.	CDH2-F	GGTGGAGGAGAAGAAGACCAG
	CDH2-R	GGCATCAGGCTCCACAGT
12.	FN-F	GAGAATGGACCTGCAAGCCCA
	FN-R	AGTGCAAGTGATGCGTCCGC
13.	VIM-F	ACCCGCACCAACGAGAAGGT
	VIM-R	ATTCTGCTGCTCCAGGAAGCG
14.	SNAI1-F	CTGGGTGCCCTCAAGATGCA
	SNAI1-R	CCGGACATGGCCTTGTAGCA
15.	SNAI2-F	TACCGCTGCTCCATTCCACG
	SNAI2-R	CATGGGGGTCTGAAAGCTTGG
16.	TWIST1-F	TGCGGAAGATCATCCCCCG
	TWIST1-R	GCTGCAGCTTGCCATCTTGGA
17.	KRT19-F	GCCACTACTACACGACCATCC
	KRT19-R	CAAACTTGGTTCGGAAGTCAT
18.	ERBB3-F	CTGATCACCGGCCTCAAT
	ERBB3-R	GGAAGACATTGAGCTTCTCTGG
19.	DDR1-F	ATGGAGCAACCACAGCTTCTC
	DDR1-R	CTCAGCCGGTCAAACTCAAACT
20.	CAV1A-F	ACAGCCCAGGGAAACCTC
	CAV1A-R	GATGGGAACGGTGTAGAGATG
